# Systematic Review of Multi-Dimensional Vulnerabilities in the Himalayas

**DOI:** 10.3390/ijerph191912177

**Published:** 2022-09-26

**Authors:** Hameeda Sultan, Jinyan Zhan, Wajid Rashid, Xi Chu, Eve Bohnett

**Affiliations:** 1 State Key Laboratory of Water Environment Simulation, School of Environment, Beijing Normal University, Beijing 100875, China; 2 Department of Environmental and Conservation Sciences, University of Swat, Mingora Swat 19130, Pakistan; 3 Department of Biology, San Diego State University, San Diego, CA 92182, USA

**Keywords:** climate change, land use/land cover change, Himalaya, PRISMA, vulnerability

## Abstract

The Himalayan region is a fragile high mountain landscape where the population experiences acute vulnerability within a complex coupled human–natural system due to environmental, social, and economic linkages. The lack of significant regional and spatial knowledge of multi-faceted vulnerabilities hinders any potential recommendations to address these vulnerabilities. We systematically reviewed the literature to recommend mitigation interventions based on the region’s socio-economic and ecological vulnerability research to date. We applied the PRISMA (Preferred Reporting of Items for Systematic Review and Meta-Analysis) criteria to search for results from four comprehensive databases. For our assessment, we compiled a final sample (*n* = 59) of vulnerability research papers to examine the vulnerability types, spatial variation, assessment methodology, and significant drivers of change. Our study represented all Himalayan countries, namely, India, Nepal, Pakistan, China, and Bhutan. More than half of the vulnerability studies were conducted in the central Himalayan region, a quarter in the western Himalayas, and a few in the eastern Himalayas. Our review revealed that the primary drivers of change were climate change, land use/land cover, and glacial lake formation. The vulnerability assessments in the Himalayan region primarily used social science methods as compared to natural science methods. While the vulnerability studies seldom assessed mitigation interventions, our analysis identified fourteen recommendations. The recommended interventions mainly included policy interventions, livelihood improvement, and adaptation measures. This study emphasized that sustainable development requires cross-sectoral interventions to manage existing resources and mitigate the confronting vulnerabilities of the region.

## 1. Introduction

Current research trends in global environmental change in the Himalayan region have improved our understanding of the complex human–natural systems that under-lie sustainability objectives. The Himalayas are one of the world’s largest mountain systems and the poorest region [1,2]. This mountainous region is also known as the “Third Pole” and the “Water towers of Asia” due to the largest glacier cover outside the polar regions [3]. The Himalayan mountain region is the source of Asia’s 10 largest rivers and supports the livelihood of around 1800 million people downstream through water provision [4]. In the Himalayan mountain region, unprecedented climate change [5,6], socio-economic change [7,8,9], high population density, poverty [10], and environmental degradation [11,12] severely threaten the lives and livelihoods of Himalayan communities. Various methods and frameworks have been used to describe and assess the vulnerability of socio-ecological systems across the Himalayas to illustrate and quantify increasing vulnerability. Fostering a refreshed dialogue about the various aspects of vulnerability, examining the social, economic, and environmental components is needed to address overarching trends, drivers, and recommendations for mitigation, from a Himalayan regional perspective. However, a systemic review is still lacking that could contribute comprehensive research synthesis to this dialogue.

Vulnerability is rooted in the study of poverty, climate impacts, and natural hazards and refers to conditions that increase a system’s susceptibility to a hazard [13,14]. Many vulnerability studies are multi-disciplinary and based on causative factors such as social, economic, and environmental factors and stressors contributing to vulnerability [15,16,17]. Vulnerability analyses seek to ask questions about these socio-ecological aspects or unravel the coupled relationships, complex linkages, and synergies operating at different spatio-temporal scales [18].

Socially disadvantaged groups, poor communities, and minorities are more vulnerable to dangerous impacts, thus enduring social aspects of vulnerability and being unable to mediate or adapt to risks and hazards [19,20]. Assessments measure social vulnerability by quantifying or evaluating food security, inequality, health, and education status [21]. Economically disadvantaged people live in cheaper, poorly built settlements, often found in areas prone to hazards, where economic well-being, income, and housing quality make people more economically vulnerable to hazards [22,23,24]. Moreover, the rural poor are the most vulnerable to hazards due to their inability to cope with disasters [25], thus becoming more vulnerable [15]. The Himalayas mainly include developing and socially vulnerable regions since a significant portion of the population consist of ethnic minorities and poverty is very high [26,27]. Due to the steady human population growth in the Himalayan region, a growing trend in vulnerability to disasters has recently been observed [28,29].

Environmental vulnerability increases because of climate change, globalization, land use/cover changes, and demographic pressures, where physical or environmental components expose humans directly to additional stressors and risks [21,30]. Climate change is one of modern-day global change’s most widely discussed drivers. It poses a severe challenge to the livelihoods of mountain dwellers [31]. Almost 12% of the world’s population live in these mountainous regions [32]; hence the issue needs more research focus. The settlements, communities, and livelihoods are increasingly unable to cope with the environmental stresses and extremes in variability caused by climate change [33,34]. As for other mountainous regions, climate change has significant ramifications for the Himalayan mountains [10]. The insufficient space in mountain areas, remote locations, and a frequent lack of communication, coupled with an increasing population, increase the environmental vulnerabilities to the risk of natural hazards, such as flooding, drought, and earthquakes [32,35,36]. With the largest glacial mass outside the polar regions, the Himalayas provide downstream water to a burgeoning population of over 200 million and is one of the most vulnerable regions to natural and anthropogenic climate change.

Numerous studies conducted across the Himalayas have attempted to understand various factors contributing to vulnerability, in which systematic research provides a comprehensive overview of the outstanding issues and recommendations. It was hypothesized that the vulnerability is varied in different regions of the Himalayas and is projected to increase in future in the face of climatic changes and anthropogenic activities. This study attempts to present the various aspects of vulnerability in the Himalayan region through a systematic review of peer-reviewed research papers. A systematic review establishes whether the Himalayan region has generalization and consistent scientific findings throughout vulnerability studies that can be broadly identified to understand how to use the local and countrywide studies together. Using the systematic review can also highlight spatial gaps in vulnerability assessment or significant research gaps in adaptation-related studies that are crucial for meeting the needs of the Himalayan region’s population. Specifically, this review intends to investigate the spatial distribution of vulnerability studies in different countries of the Himalayan region, analyze the methodological approaches used in the papers, and document and synthesize the recommendations for optimizing the vulnerable area and mitigating vulnerability in the Himalayas.

The remainder of this paper is organized as follows. Section 2 describes the methodology applied in this research. The results and discussion are presented in Section 3 and Section 4. Section 5 concludes this study with future recommendations.

## 2. Materials and Methods

### 2.1. Study Area 

**Figure 1:**
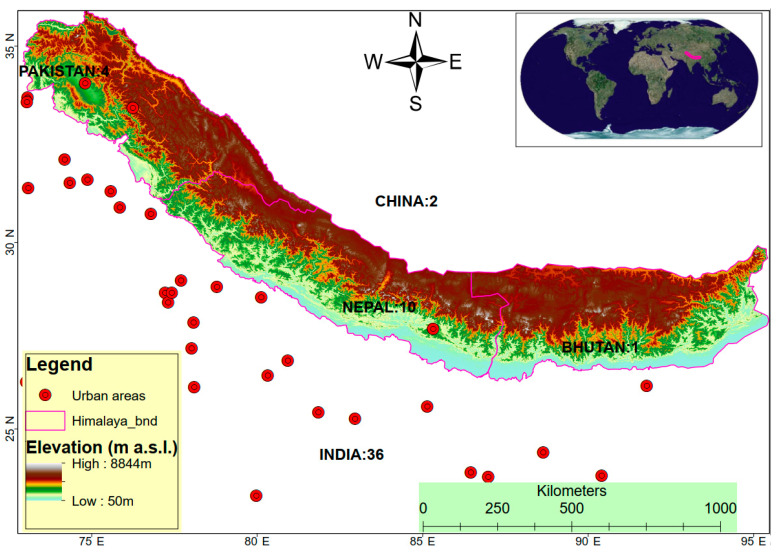
The location of the study area and the numbered distribution of the research articles on vulnerability in the Himalayan countries.

The Himalayan Mountains pass through Bhutan, China, India, Nepal, and Pakistan (Figure 1). They are the highest mountain range globally at 8849 m and are approximately 2500 km long and 160–400 km wide. The Indus–Tsangpo tectonic suture forms the northern boundary of the Himalayas on the Tibetan plateau in China [37]. Additionally, they are geologically young, seismically active, and environmentally fragile [38]. These mountains are a source of water, biodiversity, timber, mineral resources, and hydropower in the heavily populated region [39,40,41].

### 2.2. Definition of Vulnerability

The term vulnerability appeared in the 1970s during the Cold War, and its prominent advocates were scholars and practitioners concerned about the plight of third-world citizens [42,43]. Vulnerability science has considerably advanced over the last fifty years [44], mainly due to the growing susceptibility of the human population to disasters [42,45,46]. Vulnerability has evolved into a multi-disciplinary concept extensively explored in disaster management, geography, sociology, economics, and environmental studies [47,48].

Various fields apply the term vulnerability; thus, no universal and consistent definition exists [49,50]. Previous studies identified eighteen different definitions of vulnerability [46,51,52]. Since the definition of vulnerability widely varies from one discipline to another, scientists of different expertise define vulnerability according to their field of interest [49,53,54]. Research has previously defined vulnerability as “the propensity to be harmed,” or “the propensity of the exposed capital assets, physical assets, human well beings, or their livelihood to harmful hazards” [43,44,55]. Here, we define vulnerability as the potential of a system to be harmed, thereby showing sensitivity to stress or perturbation to hazards or conditions, and the capacity to respond, cope with, or adapt to the harm. 

Vulnerability is an aggregate measure of the underlying conditions [56] and is multi-dimensional [57], consisting of different types, including social vulnerability, economic vulnerability, and environmental vulnerability. However, in each facet, research has been limited to disciplinary perspectives and [58] different social, economic, and ecological stressors [59]. In the context of this paper, the term vulnerability is thus limited to the vulnerability of the human population to natural or anthropogenic stressors either directly or indirectly, although, it does not include the vulnerability of single species of flora or fauna or an object or location that has a considerable direct or indirect impact on the human population. However, it strives to explore critical gaps in vulnerability research needed for finding workable solutions.

### 2.3. Selection of the Publications for This Study

**Figure 2:**
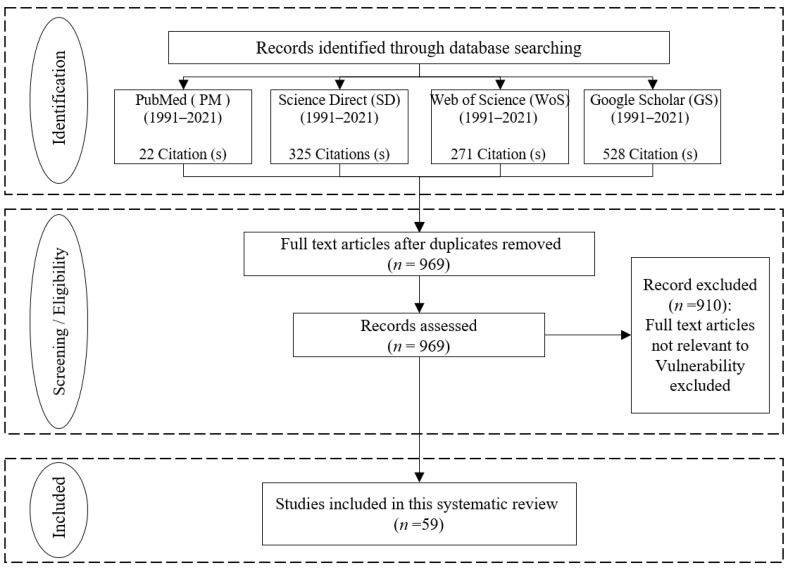
Adapted PRISMA (Preferred Reporting of Items for Systematic Review and Meta-Analysis). Note: Different phases in retrieving the published papers from the four comprehensive databases about vulnerability analysis in the Himalayan region (modified from Moher et al. [63]).

The PRISMA (Preferred Reporting of Items for Systematic Review and Meta-Analysis) methods suggest collecting data from several databases of scientific publications (Figure 2). PRISMA is widely used, extensively cited, and recognized in prominent scientific journals [60,61,62]. Specific criteria for the inclusion and exclusion of papers are adapted in the review process (Table 1). We searched for peer-reviewed papers in the comprehensive database (including Web of Science, Science Direct, Google Scholar, and PubMed) related to vulnerability in the Himalayas for a specific period (from January 1991 to December 2021). In order to be included in this review, the selected papers’ titles or keywords had to contain the term “Himalayas” or “Himalayan” and at least one of the following eleven words and phrases: vulnerability, predicting, assessment, mitigating, analyzing, earthquake, flood, climate change, landslide, glacial lakes, and seismicity. Researchers initially identified these terms using an introductory survey of the literature on vulnerability in the Himalayas (Supporting Table S1 for details).

This review includes the comprehensive results from Web of Science, Science Direct, and PubMed. However, Google Scholar provided a substantial amount of search results, where the first 100 pages were sorted for each of the search terms. Moreover, appropriate criteria were adopted to exclude non-peer-reviewed and grey literature, selecting only English language journals [64,65] (Table 1). The reasons for this exclusion are the lack of a systematic approach to examining grey literature and reliable means to verify their scientific consistency.

The literature search found numerous scientific papers in the database of Science Direct (325), Web of Science (271), Google Scholar (528), and PubMed (22). After removing duplicates, 969 articles remained. However, the final selection included a sample size of 59 papers after the rest were reviewed and the irrelevant ones removed. In this regard, the study removed papers unrelated to the Himalayas’ vulnerability or published before 1991 and after December 2021.

### 2.4. Variability Identification and Coding 

After the selection process, the total number of obtained publications (59) was finally analyzed. The papers were coded according to general categories and sorted for the variables of interest. The variables were afterward verified by the consensus of the co-authors who participated in the study, thus removing inconsistencies.

1. Spatial variation:

We identified the spatial context of the studies, i.e., which country, scale of vulnerability (local level, provincial level, national level, regional level or the entire Himalayan region), and location within the Himalayan region (western, central, or eastern). The western Himalayas lie in the Indus gorge (near Nanga Parbat in Pakistan), the Kashmir region (administered by India), Himachal Pradesh state, and the Sutlej River gorge in the east (about 550 km). Furthermore, the central Himalayas lie between the Sutlej River gorge in the west and the Arun River in the east (about 1200 km), while the eastern Himalayas lie between the Arun River gorge and the Tsangpo–Brahmaputra valley in the east (about 650 km) [66].

2. Drivers of vulnerability:

We tried to assess the type of vulnerability in the Himalayan region and its drivers, quantified the human lives and livelihoods, and documented any relevant interventions that could mitigate them.

3. Data collection method used in the sampled publications:

The data collection methods used in the sampled papers were classified as the social science method (questionnaire survey, interviews, group discussions, archives, etc.), the natural science method, (field observations, GIS/remote sensing), or a combination of the two (hybrid method).

4. Recommendations for mitigating vulnerability:

We ultimately identified the recommendations for mitigating vulnerability within the discussion or conclusion section and the result from the evaluation and/or the author’s opinion.

## 3. Results

### 3.1. Background Variables and Spatial Context

#### 3.1.1. Geographic Coverage of the Vulnerability Studies Conducted in the Himalayas

In the final 59 papers, all five countries within the Himalayan region yielded results, i.e., India (36 studies), Nepal (10 studies), Pakistan (4 studies), China (2 studies), and Bhutan (1 study), and a few regarding the whole Himalayan region (5 studies). In addition, one of the studies covered three countries (Bhutan, India, and Nepal) in the eastern Himalayas.

Three regions were investigated in this study, including the central Himalayan (35 studies), western Himalayan (12 studies), and eastern Himalayan (6 studies). The remaining four (6) studies dealt with two or more of the regions in the Himalayas (Figure 1).

#### 3.1.2. Spatial Resolution of Analysis

The level of analysis varies widely among the studied papers. Some studies were carried out at multiple levels. The studies were mainly conducted (Figure S1) at the local (40 studies), then at the provincial (10 studies), and then at the national level (5 studies) (Table S1).

#### 3.1.3. Spatial Heterogeneity of Factors 

Most of the studies in the Himalayas (57 studies) were conducted on the southern slopes of the Himalayas. In contrast, studies on the northern slopes (2 studies) of the Himalayas were rare.

**Figure 3:**
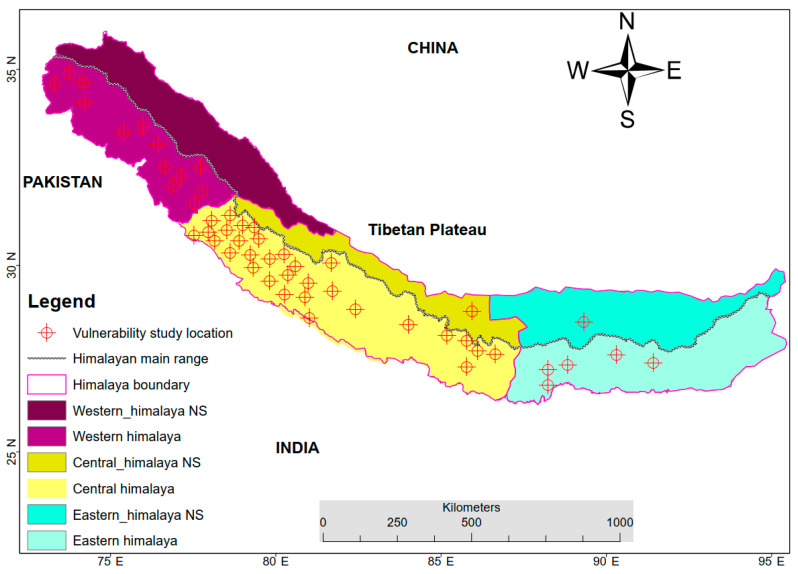
The location of the vulnerability studies. Note: The main Himalayan range divides the northern and southern parts of the Himalayas. The darker colors represent the northern slopes (NS) of the Himalayas.

In the Himalayan region, factors causing vulnerability are spatially heterogeneous. The major cities and human population centers of the Himalayas are all situated in the southern part of the Himalayas. The northern slopes of the Himalayas are sparsely populated and have no large cities. As a result, exposure and sensitivity are much higher on the southern slopes of the Himalayas (Figure 3).

### 3.2. Drivers of Vulnerability in the Himalayas 

The purpose of the vulnerability studies varied in the sampled papers. The most common objective was to quantify the vulnerability of livelihood (37.1% of the papers), then to quantify the vulnerability of the environment (25.7%), and, lastly, to document a change (15.2%), quantify the vulnerability of human health (13.3%), record an intervention (4.7%), or the vulnerability of the infrastructure (3.8%).

This research reveals that climate change was shown to be the primary driver of change in the Himalayan region (36 papers). Climate change is more significant in the Himalayan region than in other regions worldwide (Table 2). The warming rate is much higher in the Himalayan region than in other regions worldwide. In other words, the Himalayan region is more sensitive to climate change due to its fragile environment. Other causes of vulnerability in this region included land use/land cover change (5 papers). The Himalayan region’s land use/land cover is considerably changing (Table 3), followed by glacial lake formation (4 papers), which causes vulnerability in this region. Similarly, floods, poverty, and population increase (3 papers) were also found to cause vulnerability in this region. Additionally, a few papers revealed that environmental change, earthquakes, and landslides cause vulnerability in this region (Figure S2).

Most of the papers in the current review integrated two or more vulnerability types in their research (51%), a more significant number of papers in the current review were concerned with ecological vulnerability (22%) and economic vulnerability (20%). However, a smaller number of papers focused on social vulnerability (Figure S3).

**Figure 4:**
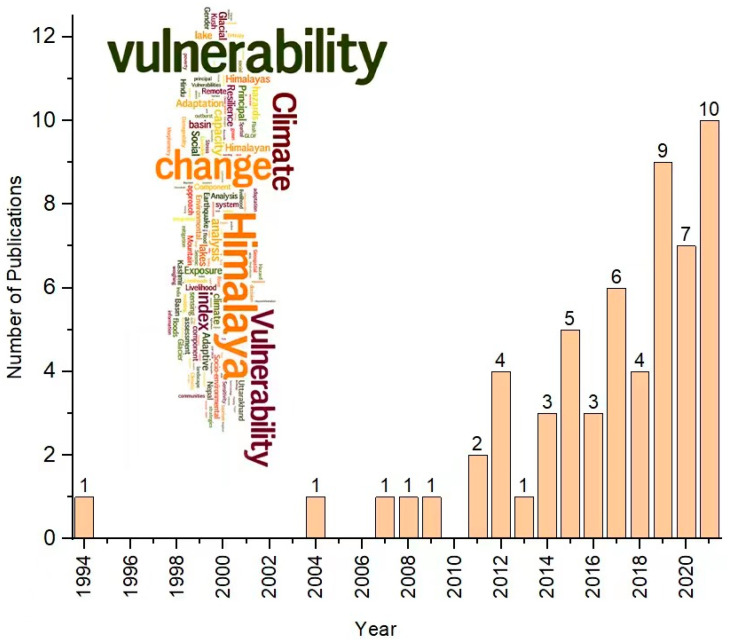
The number of peer-reviewed publications on vulnerability in the Himalayan region between 1994 and 2021. **Note:** The word cloud is based on the keywords found in the sampled papers. It was created with Wordle (http://www.wordle.net/; accessed on 20 January 2022). The word size reveals the relative frequency of their occurrence.

The sampled papers included in the systematic review were published between 1994 and December 2021. This study observes an increasing trend in the number and frequency of papers on vulnerability in the Himalayan region because only seven (7) papers were published during the first period (from 1994 to 2011), while fifty-two (52) of them were published in the last nine years (2012–2021) (Figure 4).

Furthermore, a word cloud was created based on the keywords of the sampled papers included in this review. The papers’ most common words were vulnerability, Himalaya, climate, change, glacial lake, basin, adaptation, poverty, mitigation, and analysis (Figure 4). Co-occurrences of the keywords were also illustrated through a network visualization using the VOSviewer version 1.6.18 developed by Nees Jan van Eck and Ludo Waltman at Leiden University (The Netherlands). The VOSviewer is available from https://www.vosviewer.com/download (accessed on 24 January 2022). The prominence of the words represents the strength of their co-occurrence (Figure S4).

### 3.3. Methods of Assessing and Evaluating Vulnerability Studies in the Himalayas

Methods used to collect data on vulnerability in the Himalayas varied among the sampled studies. The social science methods (44.3% of the studies) involve human responses, i.e., interviews and group discussions, while natural science methods (22.9%) study environmental factors. However, the combination of social and natural science methods was employed in 32.8% of the studies to collect vulnerability data in the Himalayas. 

The studies primarily collected data through interviews (27.8%), geographical information systems, and remote sensing (21.7%). In addition, data were gathered from secondary sources, such as archives and available databases (18.3%), field surveys (12.2%), direct observations (11.3%), and group discussions (8.7%). Some data collection methods combined a variety of methods (Table 4).

Vulnerability of human livelihoods was primarily studied in the Himalayas (47.8% of the papers), followed by environmental vulnerability (31.1%), human health vulnerability (14.4%), the vulnerability of infrastructure (5.6%), and other vulnerabilities (1.1%). Quantitative and statistical methods varied for the papers about vulnerability in the Himalayas (Figure S5).

### 3.4. Suggestions for Mitigating Vulnerability Found in the Studies 

Most studies conducted in the Himalayas recommended different measures for mitigating vulnerability. However, the assessment of mitigation measures was rarely found in those studies. The studies mainly documented recommendations without proper evaluation of the mitigation measures. On the one hand, most recommendations included policy intervention (19.7%), livelihood improvement (16.49%), adaptation measures (13.1%), monitoring (9.27%), as well as education and reducing sensitivity (8.24%), and capacity building (7.21%). Some recommendations included mitigation measures such as reducing exposure, vulnerability assessment, integrated risk management, climate-smart technologies, improved infrastructure, and government support (Table 5).

**Figure 5:**
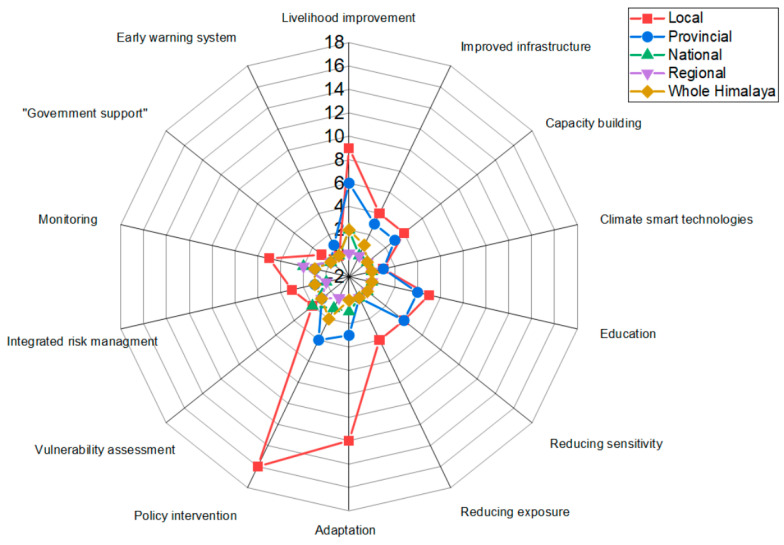
The number of studies and recommendations for vulnerability mitigation at different levels in the Himalayas.

Policy intervention was mostly recommended (24 studies) at the local level (including city and district level). Furthermore, livelihood improvement was also recommended (19 studies) at the local level (Figure 5). In contrast, climate-smart technologies and vulnerability assessment were rarely suggested at the local level. Similarly, at the provincial level, livelihood improvement (6 studies), sensitivity reduction (4 studies), and education (4 studies) were mainly recommended. However, there were comparatively fewer recommendations at the national and regional levels, including the entire Himalayan region.

**Figure 6:**
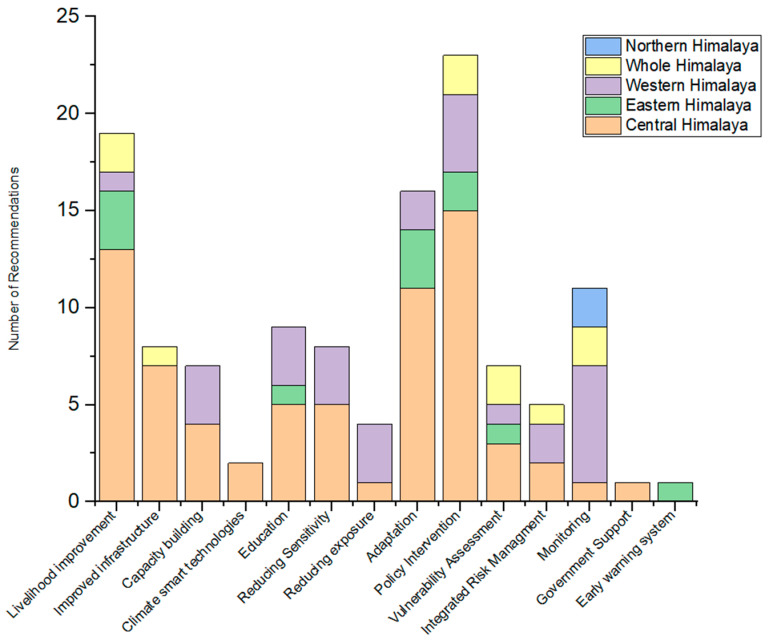
The recommendations for vulnerability mitigation for the different regions in the Himalayas.

Studies conducted in the central Himalayan region included the most significant number of recommendations, such as policy intervention (15 studies), livelihood improvement (13 studies), and adaptation (11 studies). In contrast, studies conducted in the western Himalayas had a slightly smaller number of recommendations, which included monitoring (6 studies), policy intervention (4 studies), reducing exposure (3 studies), and reduction of sensitivity (3 studies). Contrastively, recommendations of the studies about the eastern Himalayas or recommendations covering the entire Himalayan region were uncommon (Figure 6).

## 4. Discussion

This review reveals the progress of studies focused on vulnerability assessment in the Himalayan region and the critical challenges for mitigating it. There has been more attention paid to vulnerability assessment in recent years. This study showed that an increasing number of research papers were published after 2010, coinciding with the increasing vulnerability due to population growth. In the period between 1961 and 2011, the Himalayan population grew by 250%, from 19.9 to 53.8 million [66,84]. Consequently, the vulnerability of this fragile mountainous region grew with the increase in population density [85,86,87]. For example, a prior study in Nepal showed that the Climate Change Adaptation (CCA) projects for mitigating climate change vulnerability increased from just three projects in 2009 to about 30 projects in 2015, pointing towards an increasing number of CCA projects [88]. There has been more attention and a diverse number of projects attributed to vulnerability assessment in the Himalayas.

### 4.1. Geographic Coverage of the Vulnerability Studies in the Himalayas

The researchers mainly conducted vulnerability studies on the more densely populated southern slopes of the Himalayas, including Bhutan, India, Nepal, and Pakistan (Figure 4). More papers were published in India than all other studies combined, showing a skewed distribution and concentration. In contrast, vulnerability studies on the northern slopes of the Himalayas, i.e., the Tibetan region in China, were rare. 

Most vulnerability studies (59.3%) were conducted in the central Himalayan region. The central Himalayas are also the largest region, around 1200 km long, equal to the size of the western and eastern Himalayas together [66]. Moreover, they lie partly in India and Nepal [89,90]. Some studies were also conducted in the western Himalayas region (20.3%). It has the largest population of all three Himalayan regions [66]. Notably, the western Himalayan region’s glaciers are retreating faster than the central and eastern Himalayas [91]. In contrast, the eastern Himalayan region (10.1% of the studies) was relatively understudied. It consists of Nepal, India, Bhutan, and China [84] and has a sparse population [66,86]. However, the existing studies show that vulnerability increases with increasing population and density in the Himalayas [92].

About 85% of the vulnerability studies in the Himalayas were conducted in three countries: India, Nepal, and Pakistan. These countries have a significant population size [93]. Likewise, they possess social capital, research infrastructure, and technical resources to investigate vulnerability. 

### 4.2. Drivers of Vulnerability in the Himalayas

This study identified several primary drivers of vulnerability in the region, including poverty, climate change, land use and cover changes, and geological activity. The Himalayas are one of the world’s poorest regions [94]. Previous research has shown that poor and economically disadvantaged social groups, including minorities, are more vulnerable [19]. Poverty reduces their adaptability and increases their vulnerability [95,96]. 

Climate change is a significant concern for the Himalayan region’s vulnerability due to its potential social, economic, and ecological impact [28,97]. It is unknown how climate change will affect the Himalayan ecosystem [98]. Climate change has an enormous potential to affect the provision of ecosystem services by reducing agricultural production, affecting human health, and, ultimately, affecting millions of people whose livelihoods depend on natural resources [99,100,101]. Earlier studies reveal that glacial covers have retreated in the Himalayas due to global warming [102,103], making communities more vulnerable [104,105]. The livelihoods of the communities are increasingly vulnerable to climate change, reducing households’ adaptability to cope with shock, risk, and stress [50].

Changes in land use and cover are widespread throughout the Himalayan region due to the continued increase in demand and population growth. A study in the Himalayas revealed that vulnerability has increased due to deforestation, crop encroachment, and grazing [106]. Similar trends have been observed worldwide because population growth and land use/cover changes enhance the vulnerability of the inhabitants [107,108,109]. Prior studies have shown that the vulnerability of the human population is showing an increasing trend to natural hazards around the world [110,111]. In the future, vulnerability in the Himalayan region will likely increase due to the growing population and land use/cover changes as in other similar regions of the world.

The Himalayan region is geologically active, indicated by evident tectonic activities and earthquakes [37,112]. Similarly, previous studies have shown that the geotectonic activity and anthropogenic pressure are increasing yearly, enhancing vulnerability in the fragile Himalayan region [85,113,114].

### 4.3. Methods of Assessing and Evaluating Vulnerability in the Himalayas

Most of these studies primarily employed social science methods in which interviews and focus group discussions assessed vulnerability in the Himalayas. Likewise, in other regions of the world, interviews and focus groups have been used to evaluate vulnerability [115,116,117]. Many social, cultural, economic, and environmental dimensions of vulnerability [118,119,120] contribute to its type and magnitude [116,119]. Therefore, different methods have been used to assess the Himalayan region’s social, economic, and environmental vulnerabilities—nonetheless, human attitudes and social norms may cause vulnerability to remain a significant research gap.

Compared to social science methods [121], natural science methods to assess patterns of vulnerability were less employed in the studied articles. One study in the Himalayas found exposure, sensitivity, and adaptability needed to be monitored to predict and mitigate vulnerabilities [122]. However, a considerable number of studies employed the combination of social and natural science methods that develop an overall framework and vulnerability of human lives and livelihoods.

### 4.4. Recommendations for Mitigating Vulnerability Found in the Studies

This review revealed that almost half (48.4%) of the studies suggested policy intervention, livelihood improvement, and adaptation measures as the three main recommendations for mitigating vulnerability in the Himalayan region. Similarly, other studies concluded that policy, livelihood improvement, and adaptation measures are the best interventions [92,96,123,124]. In this study, policy interventions were suggested by 19.7% of studies, which implies a more significant level of policy analysis at regional and national scales be considered to address policy gaps and issues. However, a study conducted in the central Himalayas proposed agroforestry as a potential mitigation measure for climate change [125]. Interestingly, it also pointed to improving Himalayan peoples’ livelihoods as a mitigation strategy. 

Likewise, a recent study in Bhutan recommended livelihood improvement, education, and forest conservation policies to reduce vulnerability [126]. Because the Himalayas are a poor region, which adds to the vulnerability of the human population, interventions to improve livelihoods are therefore necessary to reduce vulnerability in the region. 

This review also shows that climate-smart technologies were the least recommended. Although they are now an underappreciated or understudied area, they may rise in popularity in the future. Studies have shown that technological innovation is one of the key methods in combating mankind’s present challenges, namely, climate change [127,128]. Additionally, studies conducted in other regions show that climate-smart technologies are some of the best options for mitigating vulnerabilities [129,130,131]. These technologies include climate-smart agriculture (CSA) [132,133,134], climate-smart forestry (CSF) [135,136,137], climate-smart rangeland management [138,139], and climate-smart livestock production [138,140]. CSA includes agroforestry, cover cropping, integrated pest management, traditional organic composting, integrated crop–animal farming, crop rotation, and diversification [129,141]. A recent study proposed vulnerable-smart agriculture (VSA) as replacement for CSA because the latter technique neglects an essential element, namely small-scale farmers [142].

A study by Bhattarai, et al. [143] suggested an ecosystem-based adaptation approach (EbA) that utilizes ecosystem services to reduce human vulnerability to climate change by improving adaptation. Furthermore, nature-based solutions (NbS) and natural climate solutions are the other possibilities for mitigating vulnerability to climate change [144,145,146,147]. These solutions involve protecting, conserving, managing, enhancing, restoring, and imitating natural ecosystems [148,149].

### 4.5. Limitation of the Study

The studied region includes all the Himalayas, covering the administrative boundaries of different countries. Some of the literature on vulnerability exists in the individual national languages of these countries. However, only papers written in English were selected for the current systematic review due to global considerations, although the inclusion of those papers may reveal more concerns. Although those papers were excluded from this systematic review [64], future research may seek to include them. In addition, papers published in the last thirty years (from January 1991 and December 2021) were included in the current systematic review.

## 5. Conclusions

This study concluded that demographic growth, climate change, land use/land cover change, and other natural or human changes make the Himalayas a vulnerability hotspot. The combined effect of natural and human pressures amplifies the vulnerability of this region. For example, climate change is the main recognized driver of change in the Himalayan region, where researchers are continually trying to understand adverse impacts and adaptation strategies for mitigation, such as climate-smart technologies. 

Research on vulnerability in the Himalayas varies depending on the location. The southern slopes of the Himalayas (lying in Bhutan, India, Nepal, and Pakistan) have a large population and, thus, greater vulnerability. In contrast, the Himalayan part of Tibet in China is less vulnerable due to the scarcity of its population. As a result, studies on vulnerability conducted on the northern slopes of the Himalayas are also rare, resulting in a significant research gap in understanding these areas’ vulnerabilities. Moreover, the abundance of small-scale geographic vulnerability studies at a local and provincial level suggests that this knowledge cannot be extrapolated to the heterogeneous region of the Himalayas. The Himalayas require an integrated and comprehensive vulnerability assessment to present a complete summary of the region. Therefore, a standardized or participatory evaluation can eventually lead to effective and comprehensive strategies to mitigate the vulnerability. Likewise, policy interventions can contribute to the sustainability of this populous region that suit the generalizations identified in this study. 

Although most of the studies included in this review address the main drivers of vulnerability, i.e., climate change, poverty, land use/cover change, topography, etc., recommendations to adapt to vulnerabilities in the Himalayas are infrequent. They, therefore, call for appropriate attention in some future research. Climate-smart technologies and nature-based solutions to mitigate vulnerability in the Himalayas require adequate attention for further research. However, human attitudes and social norms that may cause vulnerabilities have not yet been subjected to in-depth research and thus remain a critical research gap.

## Figures and Tables

**Table 1:** The criteria for the inclusion and exclusion of scientific papers in the review process.

Criteria	Included	Excluded
Publication date	Articles published between January 1991 and December 2021	Articles published before 1991 or after December 2021
Document type	Peer-reviewed articles	Grey literature, book chapters, conference proceedings, reports, notes
Study region	Bhutan (entire country) China (part of the Tibetan autonomous region) India (the Himalayas passing through the provinces in the northern region) Nepal (entire country) Pakistan (parts of Gilgit Baltistan, Khyber Pakhtunkhwa, and Azad Kashmir regions in the Himalayas)	Other parts of China, India, and Pakistan not lying in the Himalayan mountainous region
Language Theme of the current study	English language articles only Articles conducted on vulnerability to hazards, including social, economic, and environmental vulnerabilities.	Articles in other languages, including national or regional languages Articles not explicitly related to vulnerabilities
Databases for the article search	Google Scholar, Science Direct, PubMed, and Web of Science	Articles not available in these comprehensive databases

**Table 2:** Climate warming rates from the selected sources in the Himalayan region.

Location	Period	Warming Rate	Reference
Global Mean Surface Temperature	1951–2012	0.12 °C/decade	[67,68]
Himalayan region Kashmir Himalayas (India)	1982–2006 1980–2016	0.60 °C/decade 0.24 °C/decade	[69] [70]
Hindukush Himalaya (Pakistan) Hindukush, Karakoram, Himalayan region (Pakistan)	1986–2010 1986–2015	0.39 °C/decade 0.25 °C/decade	[71] [72]
Himalayan region (India)	1990–2016	0.72 °C/decade	[73]
Trans-Himalaya region (Nepal) Himalaya region (Bhutan) Himalaya region (Bhutan) Himalayan region (China)	1977–1994 1997–2017 1985–2002 1991–2007	0.90 °C/decade 0.38 °C/decade 0.30 °C/decade 0.73 °C/decade	[74] [75] [69] [76]

**Table 3:** Land use/land cover changes in the Himalayan region.

Country/ Location	Type of Land use/Landcover Changes	Period	Source
Himalayas (Bhutan)	Increase in forest cover	1990–2010	[77]
Himalayan region (China)	Glaciers decreased Increased glacier lakes formation	1990–2015	[78]
Central Himalayas, (India) Kashmir Himalayas (India)	Increasing deforestation, forest fragmentation Cropland decreased	1976–2006 1990–2017	[79] [80]
Himalayas (Nepal)	Deforestation Forest degradation	1976–2001	[81]
Hindukush Himalaya (Pakistan) Himalayan region (Pakistan)	Built-up area increased Cropland increased Built-up area increased Vegetation cover decreased	2008–2018 1990–2017	[82] [83]

**Table 4:** Ranking the types of vulnerabilities assessed through different data collection techniques.

Data Collection	Number of Articles	Main Type of Vulnerability Assessed			More than One Type of Vulnerability
		Environmental Vulnerability	Economical Vulnerability	Social Vulnerability	
Interviews	32 (27.8%)	4	8	3	17
Geographical Information System and remote sensing	25 (21.7%)	10	2	0	13
Archives and available databases	21 (18.3%)	6	2	1	12
Field surveys	14 (12.2%)	2	4	1	7
Group discussions	13 (11.3%)	3	4	0	6
Direct observation	10 (8.7%)	0	4	2	4
Overall	115 (100%)	14	12	8	59

**Table 5:** Recommendations from the papers included in this systematic review.

S. No	Recommendation	Percentage
1	Policy intervention	19.7%
2	Livelihood improvement	15.6%
3	Adaptation measures	13.1%
4	Monitoring measures	9.0%
5	Education	7.4%
6	Reducing sensitivity	6.6%
7	Improved infrastructure	6.6%
8	Vulnerability assessment	5.7%
9	Capacity building	5.7%
10	Integrated risk assessment	4.1%
11	Reducing exposure	3.3%
12	Climate-smart technologies	1.6%
13	Government support	0.8%
14	Early warning system	0.8%

## Data Availability

The datasets generated during and/or analysed during the current study are available from the corresponding author on reasonable request.

## Supplementary Materials

The following supporting information can be downloaded at: https://www.mdpi.com/article/10.3390/ijerph191912177/s1, Figure S1: Vulnerability studies in the Himalayas by the level of analysis. Figure S2: Number of articles classified by category of the vulnerability identified. Figure S3: The type of vulnerability focused on in the articles included in this review. Figure S4: The keyword co-occurrence analysis (in Scopus) related to vulnerability in the Himalayas. Figure S5: Methods used in the studied articles on vulnerability in the Himalayas. Table S1: Vulnerability data for the analysis.
