# Supplementary material for: Systematic Review of Multi-Dimensional Vulnerabilities in the Himalayas
Source: Int J Environ Res Public Health. 2022 Sep 26;19(19):12177. doi: 10.3390/ijerph191912177 (PMC9566038; doi:10.3390/ijerph191912177)
Supplement: Supplementary file 1 [file ijerph-19-12177-s001.zip › ijerph-1879507-supplementary/ijerph-1879507-supplementary-done.pdf]

International Journal of
Environmental Research and Public Health

Supplementary Information

Systemic Review of Multi-Dimensional Vulnerabilities in the Himalayas

Hameeda Sultan [1], Jinyan Zhan [1,*], Wajid Rashid [2], Xi Chu [1] and Eve Bohnett [3]

[1] State Key Laboratory of Water Environment Simulation, School of Environment, Beijing Normal University, Beijing 100875, China
[2] Department of Environmental and Conservation Sciences, University of Swat, Mingora Swat 19130, Pakistan
[3] Department of Biology, San Diego State University, San Diego, CA 92182, USA
* Correspondence: zhanjy@bnu.edu.cn

Supplementary Figures

Figure S1: Vulnerability studies in the Himalayas by the level of analysis.
Figure S2: Number of articles classified by category of the vulnerability identified.
Figure S3: The type of vulnerability focused on in the articles included in this review.
Figure S4: The keyword co-occurrence analysis (in Scopus) related to vulnerability in the Himalayas.
Figure S5: Methods used in the studied articles on vulnerability in the Himalayas.

Supplementary Table

Table S1. Vulnerability data for the analysis.

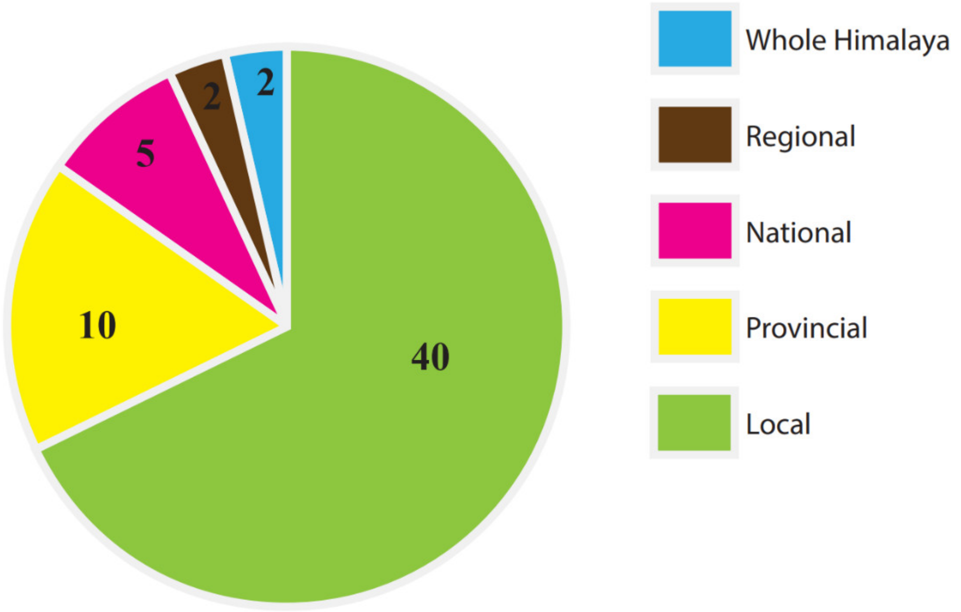

Figure S1. Vulnerability studies in the Himalayas by the level of analysis.

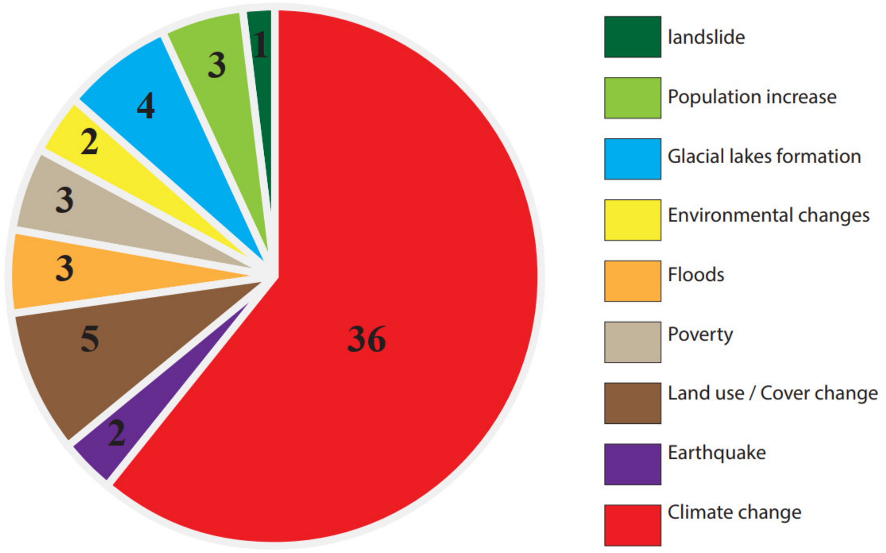

Figure S2. Number of articles classified by category of the vulnerability identified.

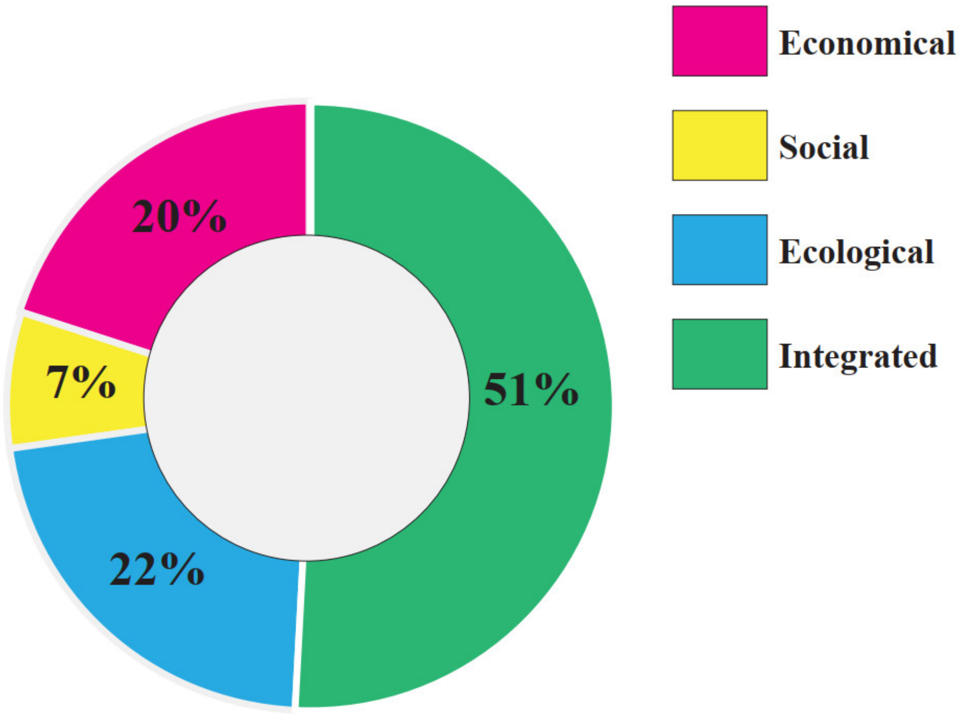

Figure S3. The type of vulnerability focused on in the articles included in this review.

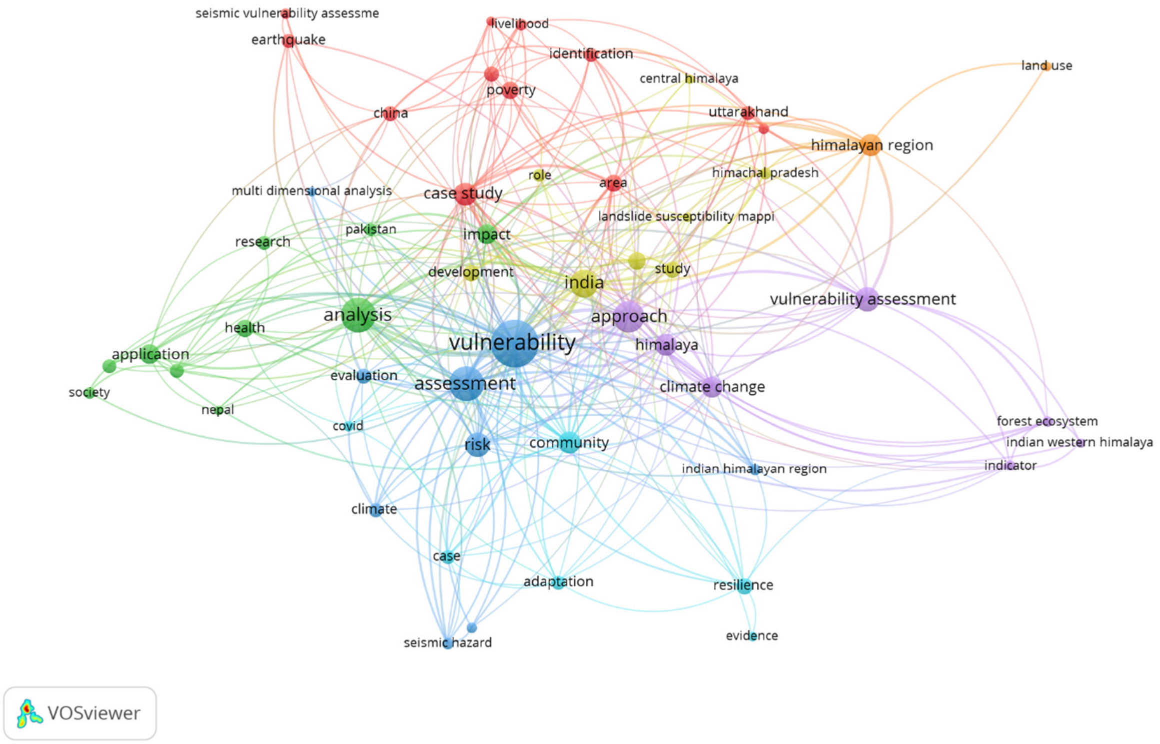

Figure S4. The keyword co-occurrence analysis (in Scopus) related to vulnerability in the Himalayas.

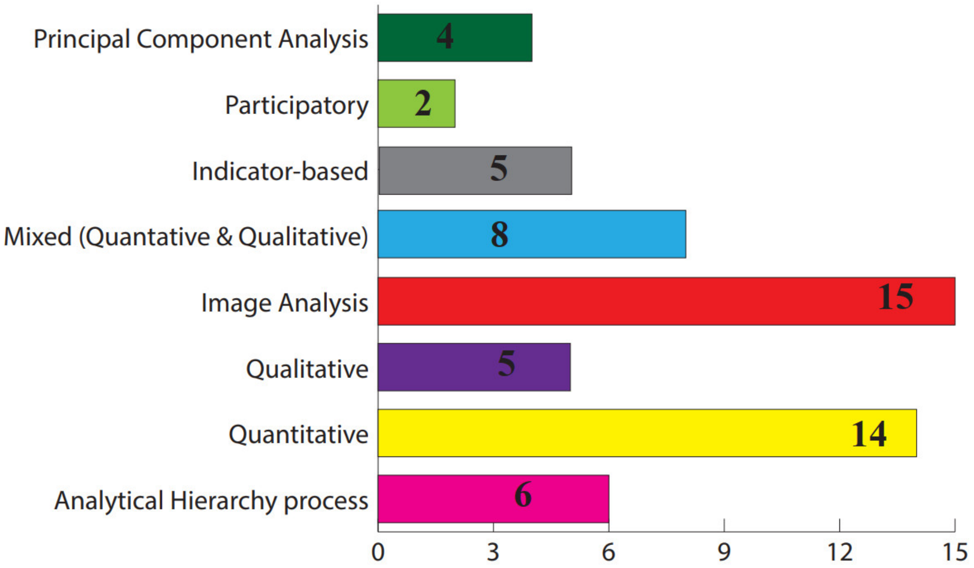

Figure S5. Methods used in the studied articles on vulnerability in the Himalayas.
